# Trait Reward Sensitivity Modulates Connectivity with the Temporoparietal Junction and Anterior Insula during Strategic Decision Making

**DOI:** 10.1101/2023.10.19.563125

**Published:** 2024-03-18

**Authors:** Daniel Sazhin, James B. Wyngaarden, Jeff B. Dennison, Ori Zaff, Dominic Fareri, Michael S. McCloskey, Lauren B. Alloy, Johanna M. Jarcho, David V. Smith

**Affiliations:** 1 Department of Psychology & Neuroscience, Temple University, Philadelphia, PA, USA; 2 Derner School of Psychology, Adelphi University, Garden City, NY, USA

**Keywords:** Reward Sensitivity, Strategic Behavior, Ultimatum Game, Dictator Game, Connectivity

## Abstract

Many decisions happen in social contexts such as negotiations, yet little is understood about how people balance fairness versus selfishness. Past investigations found that activation in brain areas involved in executive function and reward processing was associated with people offering less with no threat of rejection from their partner, compared to offering more when there was a threat of rejection. However, it remains unclear how trait reward sensitivity may modulate activation and connectivity patterns in these situations. To address this gap, we used task-based fMRI to examine the relation between reward sensitivity and the neural correlates of bargaining choices. Participants (N = 54) completed the Sensitivity to Punishment (SP)/Sensitivity to Reward (SR) Questionnaire and the Behavioral Inhibition System/Behavioral Activation System scales. Participants performed the Ultimatum and Dictator Games as proposers and exhibited strategic decisions by being fair when there was a threat of rejection, but being selfish when there was not a threat of rejection. We found that strategic decisions evoked activation in the Inferior Frontal Gyrus (IFG) and the Anterior Insula (AI). Next, we found elevated IFG connectivity with the Temporoparietal junction (TPJ) during strategic decisions. Finally, we explored whether trait reward sensitivity modulated brain responses while making strategic decisions. We found that people who scored lower in reward sensitivity made less strategic choices when they exhibited higher AI-Angular Gyrus connectivity. Taken together, our results demonstrate how trait reward sensitivity modulates neural responses to strategic decisions, potentially underscoring the importance of this factor within social and decision neuroscience.

## Introduction

Social situations such as negotiations often require people to strategically consider social norms while minimizing their threat of being rejected. It is understood that people act fairly when they could be rejected in the Ultimatum Game (UG; Güth et al., 1982; Wells & Rand, 2013) and selfishly when there is not a threat of rejection in the Dictator Game (DG; Engel, 2011; Kahneman et al., 1986). Thus, people exhibit strategic behavior by making smaller contributions in the DG than in the UG (Charness & Gneezy, 2008). Past investigations suggested there are relations between strategic behavior and measures of social functioning such as emotional intelligence (Kench et al., 2007) and Machiavellianism (Spitzer et al., 2007). A possible explanation for strategic behavior is the social heuristics hypothesis, which suggests people share more or less intuitively based on self-interest, and greater deliberation yields more strategic choices (Rand, 2016; Rand et al., 2016). In addition to social information processing, empathy can increase sharing in the Dictator Game (Klimecki et al., 2016), suggesting that empathetic experience can result in more consistent offers between DG and UG.

Strategic decisions have also been associated with brain activation in the ventral striatum (VS), dorsal lateral prefrontal cortex (dlPFC), and lateral orbitofrontal cortex (OFC) (Spitzer et al., 2007). Other work has implicated dorsal anterior cingulate cortex (dACC) and posterior cingulate cortex (PCC) (Weiland et al., 2012) . Decisions made in social contexts reliably elicited activation in the right temporoparietal junction (rTPJ) (Behrens et al., 2008; Carter et al., 2012; Dennison et al., 2022), and greater contributions in the DG (Gianotti et al., 2018; Morishima et al., 2012). Finally, stimulating the right dlPFC resulted in fairer offers in the UG versus the DG (Knoch et al., 2006; Ruff et al., 2013; Strang et al., 2015) and disruption of the right dlPFC resulted in higher offers in the DG (Zinchenko et al., 2021). In sum, it is known that brain activation can distinguish some strategic decision making in social contexts.

Relatively less is known about how task-based brain connectivity (Friston et al., 1997) modulates strategic decisions. Past research suggests that signals related to the receipt of rewards are encoded through corticostriatal connectivity (D. V. Smith, Rigney, et al., 2016) Next, DG decisions modulated VS-TPJ connectivity (Park et al., 2017) and dorsal striatum-lateral PFC connectivity (Crockett et al., 2017). Since past findings suggested that the VS responses were related to strategic learning (Zhu et al., 2012) and were elevated with greater strategic decision making (Spitzer et al., 2007), corticostriatal connectivity may be modulated by social contexts. Individual differences in trait reward sensitivity may affect how people make social valuations, possibly moderating neural connectivity in social contexts. Reward sensitivity has been studied as a clinical measure (Alloy et al., 2016; Carver & White, 1994; Nusslock & Alloy, 2017), revealing that people who are hyper and hyposensitive to rewards are at risk for substance use and bipolar or depressive disorders (Bart et al., 2021). However, little is known about how corticostriatal connectivity is modulated by reward sensitivity (Sazhin et al., 2020). For instance, people who are more sensitive to rewards may overvalue their initial endowment in UG and DG contexts and may be loath to share it with a stranger.

Since reward sensitivity is associated with risky behavior (Scott-Parker & Weston, 2017), higher Machiavellianism (Birkás et al., 2015), and with more strategic behavior (Scheres & Sanfey, 2006), it is plausible that reward sensitivity may modulate brain responses while making strategic decisions. With this interpretation, reward sensitivity could potentially increase activation or connectivity from reward areas such as the ventral striatum to modulate strategic decisions. Evidence supporting this interpretation would suggest that strategic decisions may be mechanistically driven by reward processing and that reward sensitivity is a reflection of bottom-up reward responses. Alternatively, cognitive processes involved in attention and social decision making such as the TPJ may be affected while making strategic decisions. Evidence supporting this interpretation would suggest that top-down cognitive processes may be modulated by trait reward sensitivity while making strategic decisions. Overall, examining the role of reward sensitivity and brain responses during strategic decisions could unpack reward, attentional, or value-based decision-making mechanisms that facilitate overcoming social heuristics and thereby acting on self-interest.

Since the striatal response is sensitive to social valuation (Chein et al., 2011; Fareri & Delgado, 2014), examining how social context is modulated by trait reward sensitivity and striatal responses could help unravel how aberrant reward processing may result in maladaptive decisions that can contribute to substance use (Dalley & Robbins, 2017), or possibly diminished strategic behavior in social situations. Thus, our aims in this investigation were to assess how brain activity and connectivity are modulated by one’s strategic decisions and by trait reward sensitivity. Using functional magnetic resonance imaging (fMRI), we administered Ultimatum and Dictator Games to participants to investigate associations between strategic behavior, reward sensitivity, and brain connectivity while controlling for substance use. We examined activation patterns during both endowment and decision phases, corticostriatal connectivity during the decision phase, and how these patterns were modulated by strategic behavior and reward sensitivity.

To examine these questions, we assessed several pre-registered hypotheses (https://aspredicted.org/55gd8.pdf). We expected greater activation of the VS and vmPFC during the endowment of money, and that reward sensitivity positively modulates activation in the VS and vmPFC. Such activation during endowment would suggest that reward receipt is modulated by reward sensitivity. Next, we investigated activation within the dlPFC, ACC, SPL, IPS, vmPFC, VS, and TPJ during each task condition (DG-P, UG-P, and UG-R), and specifically to strategic decisions (UG-P > DG-P). We expected that the dlPFC would exhibit stronger activation in response to strategic decisions. These findings during the decision phase would suggest that changes in social context with respect to norm compliance evoke differential activation in the brain. Finally, we expected to find elevated ventral striatal responses to strategic behavior (UG-P > DG-P) during the decision phase to be associated with enhanced effective connectivity with regions modulated by social information (e.g., vmPFC, mPFC, and TPJ). In addition, we expected that these neural effects would be enhanced in individuals with higher level of self-reported reward sensitivity. Such findings would suggest that reward sensitivity is an important dimension of understanding brain responses associated with strategic behavior.

Our analyses focus on two key questions. First, how do strategic decisions in social situations modulate brain activation and connectivity? Second, how does trait reward sensitivity modulate brain connectivity while making strategic decisions? Assessing neural connectivity during strategic decisions and how reward sensitivity modulates these processes would 1) improve our understanding of the mechanisms of how people cooperate and defect in social situations, and 2) help determine how aberrant patterns of reward sensitivity may be a risk factor for maladaptive social decision making.

## Materials and Methods

### Participants

Although in our pre-registration (https://aspredicted.org/55gd8.pdf) we specified that we would collect imaging data from 100 participants (18–22) (Sazhin et al., 2020), we ultimately recruited 59 participants (D. V. Smith et al., 2024) due to constraints imposed by the COVID-19 pandemic. We excluded a total of 5 participants for our neuroimaging analyses based on our pre-registered criteria and missing data. Specifically, three participants were excluded due to failure to respond during behavioral tasks, where there were greater than 20% missing responses on a given run. One participant was excluded due to incomplete behavioral data. One participant was excluded due to issues with data collection. Three of the 54 participants had one of the two task runs excluded due to excessive head motion. Our final neuroimaging sample resulted in 54 participants (mean age: 20.95 years, SD: 1.78 years; 24.1% male). Several behavioral analyses related to social functioning had a more limited sample due to missing data. Specifically, 9 participants were missing behavioral data related to social functioning, resulting in a sample of 45 participants (mean age: 20.74 years, SD: 1.54 years; 24.4% male) for several behavioral analyses. All participants were compensated at a rate of $25 per hour inside the scanner and $15 per hour outside the scanner, and received bonuses based on their decisions, resulting in a total payment ranging from $140 to $155. Participants were recruited using Facebook advertisements and fliers posted around the Temple University campuses. We verified that participants were eligible to be scanned using fMRI by the following criteria: a) not being pregnant, b) free of major psychiatric or neurologic illness, and c) not under the influence of substances as evidenced by a breathalyzer test and urine drug screen. All the participants provided written informed consent as approved by the Institutional Review Board of Temple University.

### Procedure

Potential participants were identified based on their responses to an online screener questionnaire using the SONA research platform that assessed reward sensitivity using the Behavioral Activation Subscale (BAS; Carver & White, 1994) and the Sensitivity to Reward subscale (SR; Torrubia et al., 2001). Using methods consistent with our prior work (e.g., Alloy, Bender, et al., 2009), we compared results between both SR and BAS to ensure that participants were responding consistently and truthfully by excluding participants with scores that were less than +/−1 quintile on both subscales. Participants also were called on the phone and asked to abstain from alcohol or drug usage for 24 hours prior to the scan. Participants were excluded if they reported that they took any psychoactive medications. Participants attended two appointments, consisting of a battery of psychometric surveys, and a mock scan, followed by a second appointment consisting of the fMRI scan and behavioral tasks.

### Individual Difference Measures

Reward Sensitivity. To measure reward sensitivity, we used the Behavioral Activation Scale (BAS; Carver & White, 1994) and the Sensitivity to Punishment/Sensitivity to Reward Questionnaire Reward subscale (SPSRWD; Torrubia et al., 2001)). The BAS is a 20-item self-report questionnaire that measures sensitivity to appetitive motives. The SPSRWD is a 24-item self-report measure that assesses how people feel in response to rewarding stimuli.

#### Substance Use.

To measure substance use among healthy adult individuals, we used the Alcohol Use Disorders Identification Test (AUDIT; Babor et al., 1992) and the Drug Use Identification Test (DUDIT; A. Berman et al., 2003; A. H. Berman et al., 2005). The AUDIT is a 10-item self-report measure that assesses frequency of usage over the past year and the self-reported extent to which alcohol use affects the person’s life. The DUDIT scale is an 11-item self-report measure counterpart of the AUDIT that assesses frequency and disruptiveness of non-alcohol related substance use. DUDIT contains references to a wide array of substances, including marijuana, cocaine, and others.

#### Social Functioning.

To measure social functioning, we measured trait emotional intelligence and attitudes toward rejection. The trait Emotional Intelligence (EI) questionnaire (TEIQe) is a 30-item self-report measure that assesses individual differences in trait empathy, emotion regulation and perspective taking in emotional contexts (Petrides, 2009). Attitudes toward reciprocity were investigated through the 9-item punishment sub-scale of the Personal Norms of Reciprocity (PNR) measure (Perugini et al., 2003).

### Experimental Design

**Figure 1.**
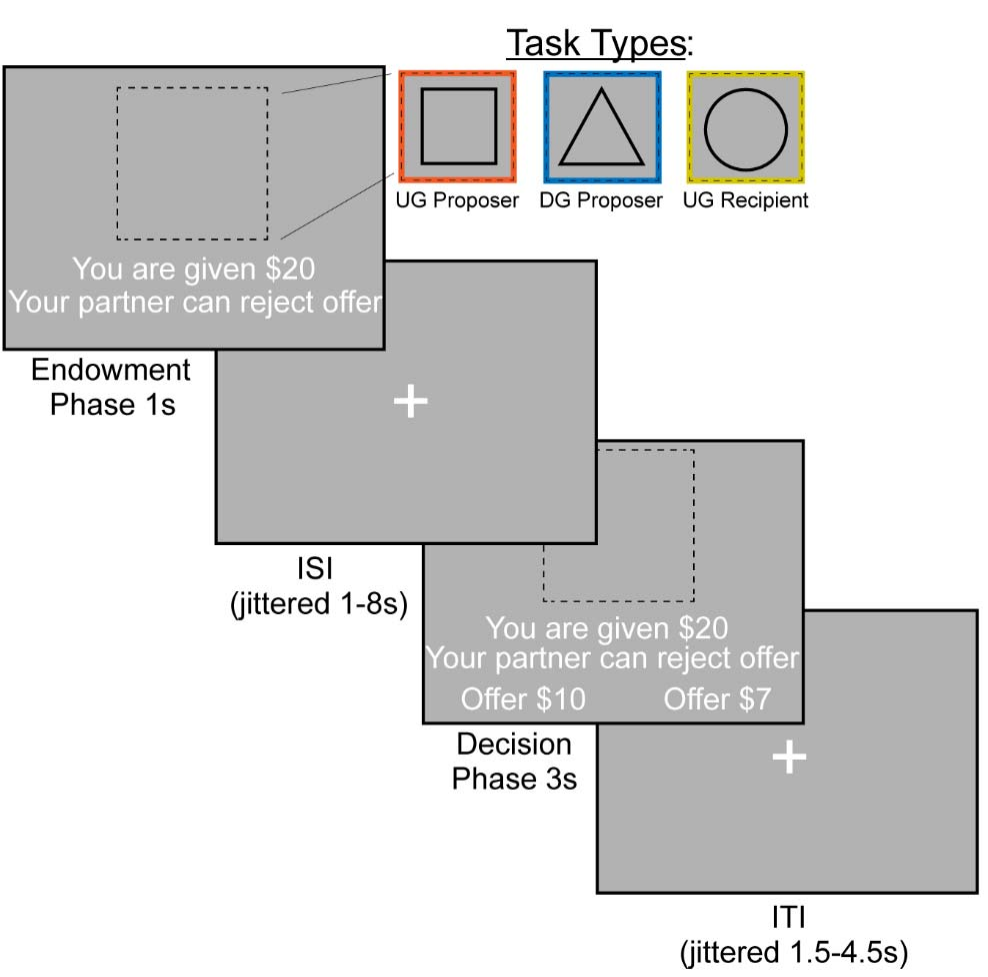
FMRI-based Bargaining tasks to Measure Strategic Behavior Using the Dictator and Ultimatum Games. We operationalized strategic behavior as offering more in the Ultimatum Game and less in the Dictator Game, as this strategy would maximize earnings. During the Endowment phase, the participant learned how much money they were given and which task they would complete. A square indicated that the participant would be acting as the Proposer in the Ultimatum Game or deciding how much money to split with a counterpart. A triangle indicated that the participant would act as the Proposer in the Dictator Game. Finally, a circle indicates that the participant would be the Recipient in the Ultimatum Game, which allowed them to decide whether they would accept or reject an offer given to them. We included the Recipient condition so that participants buy into the manipulation of the threat of punishment during the Ultimatum Game as a proposer. During the Decision Phase, the participant as a proposer decided to offer More or Less to their counterpart. As a recipient, whether to accept or reject the offer.

We examined bargaining behavior using the Ultimatum (Figure 1) (Güth et al., 1982) and Dictator Games (Figure 1) (Kahneman et al., 1986) (~15 min, counterbalanced across participants). In the Dictator Game (DG), the participant decided how much of an endowed sum ($15–25) to share with their partner. To ensure that participants were deceived into believing that their decisions had a social impact, the participant was told their partner was represented by decisions made by past participants in the study, and that their decisions would be used with future participants. In addition, each decision was made by a different partner, resulting in each trial being a one-shot game. This design is used to minimize the concern for reciprocity, reputation or other motives beyond social preferences for fairness while making each choice (Yamagishi et al., 2012). In the Ultimatum Game (UG), participants acted as the proposer in some trials and the responder in other trials. As the proposer, participants chose a split of their endowment; however, they were aware that their counterpart could reject their offer. As a recipient in the UG, participants were presented offers from partners that they could choose to accept or reject. If they chose to reject the offer, neither they nor the proposer made any money for that trial. Although our hypotheses and analyses were not focused on the recipient decisions, we included this condition to make the task more believable by making participants think that their unfair proposals could be rejected. We characterize strategic behavior as behavior that offers lower amounts in DG and generally higher amounts in UG, as this strategy would maximize earnings and minimize the threat of rejection.

The experiment consisted of three conditions (Dictator Game- Proposer (DG-P), Ultimatum Game- Proposer (UG-P), Ultimatum Game- Recipient (UG-R)) that were presented in a counterbalanced order. The tasks were administered using PsychoPy (Peirce et al., 2019) across two 7:30 minute runs. Each run consisted of 36 trials, with 12 trials in each condition. On each trial, the participant was endowed with a sum of money between $15–$25. The participant experienced an interstimulus interval (ISI) of 1.5–4.5 seconds, M = 2.42s. This was followed by an indication of the type of trial the participant is playing through a target stimulus. If they were acting as the proposer in the DG, they were presented with a triangle. If they were acting as a proposer in the UG, they were presented with a square. Finally, if they were acting as a recipient in the UG they were presented with a circle. During the decision phase as proposer, participants are presented with the option to select a More or Less split. During the decision phase as a recipient, participants have the choice whether to accept or reject the offer. If a participant missed a trial, the screen indicated that they were too slow and recorded a missed trial in the log. Subsequent to each trial, there was a variable duration intertrial interval of 1–8 seconds; M = 2.7s.

### Behavioral Analysis

To examine whether participants acted strategically through offering more as a Proposer in the Ultimatum Game condition versus the Dictator Game condition, we used a mixed effect linear model. The regressors included the task (UG-P or DG-P), trial endowment, and the proportion of the endowment the participant offered. While we included the recipient condition (UG-R) so that participants experience offers to understand the threat of punishment as proposers, our main questions do not assess recipient behavior. Nonetheless, as a manipulation check to check whether participants rejected unfair offers (i.e., offers with a proportion substantially less than half of the endowment) in the Ultimatum Game as a recipient, we used a logistic linear regression model. Next, we assessed whether there were associations between decisions and measures of social functioning, reward sensitivity, and substance use. Given that both hyper- and hypo-sensitivity to rewards have been linked to substance use (Alloy et al., 2009; Bart et al., 2021; Franken & Muris, 2006), we control for levels of substance use in our data while assessing reward sensitivity. We used correlations between trait measures with the proportions offered in the UG versus DG (i.e., Spitzer et al., 2007). This method of measuring strategic behavior was used rather than pooling hypothetical total earnings (see deviations from pre-registration) as this method avoided inferring earnings and simply used the participants’ decisions. We also conducted exploratory analyses to 1) assess whether there are associations between strategic behavior and reward sensitivity and substance use, and 2) whether there are associations between the individual difference measures and individual conditions (DG-P, UG-P, and UG-R).

We conducted analyses on the included self-report measures to ensure that they were correctly operationalized for further analyses. Since the BAS and SR subscale of the SPSRWD were highly correlated *r*(52) = .71, *p* < .001, we combined them into a single composite measure of reward sensitivity using their combined z-scores. Reward sensitivity scores were binned into deciles to produce an even distribution for subsequent analysis. Finally, because both hyper- and hypo-sensitivity to rewards have been linked to substance use (e.g., Alloy et al., 2009; Bart et al., 2021; Franken & Muris, 2006), we squared the binned composite reward sensitivity scores to create an additional, quadratic measure of aberrant reward sensitivity. In other words, aberrant reward sensitivity explores whether there are consistent patterns across people who are either high or low in reward sensitivity. Next, we found that AUDIT and DUDIT also were correlated *r*(52) = .32, *p* = .02. As a result, we operationalized problematic substance use through z-scoring the responses between the measures and combining them into a single composite z-score of problematic substance use using the same method as described for reward sensitivity.

### Neuroimaging Data Acquisition

Functional images were acquired using a 3T Siemens PRISMA MRI scanner. Bold Oxygenation Level-Dependent (BOLD) sensitive functional images were acquired using a gradient echo-planar imaging (EPI) sequence (240 mm in FOV, TR = 1,750 ms, TE = 29 ms, voxel size of 3.0 × 3.0 × 3.0 mm3, flip angle = 74°, interleaved slice acquisition). Each of the two runs included 225 functional volumes. We also collected single-band reference images with each functional run of multi-band data to improve motion correction and registration. To facilitate anatomical localization and co-registration of functional data, a high-resolution structural scan was acquired (sagittal plane) with a T1-weighted magnetization=prepared rapid acquisition gradient echo (MPRAGE) sequence (224 mm in FOV, TR = 2,400 ms, TE = 2.17 ms, voxel size of 1.0 × 1.0 × 1.0 mm3, flip angle 8°). In addition, we also collected a B0 fieldmap to unwarp and undistort functional images (TR: 645 ms; TE1: 4.92 ms; TE2: 7.38 ms; matrix 74×74; voxel size: 2.97×2.97×2.80 mm; 58 slices, with 15% gap; flip angle: 60°).

#### Preprocessing of Neuroimaging Data.

Neuroimaging data were converted to the Brain Imaging Data Structure (BIDS) using HeuDiConv version 0.9.0 (Halchenko et al., 2019). Results included in this manuscript come from preprocessing performed using fMRIPrep 20.2.3 (Esteban et al., 2018, 2019), which is based on Nipype 1.4.2 (K. Gorgolewski et al., 2011; K. J. Gorgolewski et al., 2017). The details described below are adapted from the fMRIPrep preprocessing details; extraneous details were omitted for clarity. Head motion was calculated to determine exclusions. Excess head motion was defined where at least one run was a motion outlier, characterized by either fd mean exceeding 1.5 times the interquartile range above the 75th or tsnr values lower than 1.5 times the lower bound minus the 25th percentile per neuroimaging data quality measures from MRIQC).

#### Anatomical data preprocessing.

The T1-weighted (T1w) image was corrected for intensity non-uniformity (INU) with ‘N4BiasFieldCorrection’, distributed with ANTs 2.3.3, and used as T1w-reference throughout the workflow. The T1w-reference was then skull-stripped with a Nipype implementation of the àntsBrainExtraction.sh` workflow (from ANTs), using OASIS30ANTs as target template. Brain tissue segmentation of cerebrospinal fluid (CSF), white-matter (WM), and gray-matter (GM) was performed on the brain-extracted T1w using `fast` (FSL 5.0.9). Volume-based spatial normalization to one standard space (MNI152NLin2009cAsym) was performed through nonlinear registration with àntsRegistration` (ANTs 2.3.3), using brain-extracted versions of both T1w reference and the T1w template. The following template was selected for spatial normalization: ICBM 152 Nonlinear Asymmetrical template version 2009c (TemplateFlow ID: MNI152NLin2009cAsym).

#### Functional data preprocessing.

For each BOLD run, the following preprocessing steps were performed.. First, a reference volume and its skull-stripped version were generated by aligning and averaging 1 single-band references (SBRefs). A B0-nonuniformity map (or fieldmap) was estimated based on a phase-difference map calculated with a dual-echo GRE (gradient-recall echo) sequence, processed with a custom workflow of SDCFlows inspired by the èpidewarp.fsl` script (http://www.nmr.mgh.harvard.edu/~greve/fbirn/b0/epidewarp.fsl) and further improvements in HCP Pipelines. The fieldmap was then co-registered to the target EPI (echo-planar imaging) reference run and converted to a displacements field map (amenable to registration tools such as ANTs) with FSL’s `fuguè and other SDCflows tools. Based on the estimated susceptibility distortion, a corrected EPI (echo-planar imaging) reference was calculated for a more accurate co-registration with the anatomical reference. The BOLD reference was then co-registered to the T1w reference using `flirt` (FSL 5.0.9) with the boundary-based registration cost-function. Co-registration was configured with nine degrees of freedom to account for distortions remaining in the BOLD reference. Head-motion parameters with respect to the BOLD reference (transformation matrices, and six corresponding rotation and translation parameters) are estimated before any spatiotemporal filtering using `mcflirt`.

BOLD runs were slice-time corrected using `3dTshift` from AFNI 20160207. First, a reference volume and its skull-stripped version were generated using a custom methodology of fMRIPrep. The BOLD time-series (including slice-timing correction when applied) were resampled onto their original, native space by applying a single, composite transform to correct for head-motion and susceptibility distortions. These resampled BOLD time-series will be referred to as preprocessed BOLD in original space, or just preprocessed BOLD.

The BOLD time-series were resampled into standard space, generating a preprocessed BOLD run in MNI152NLin2009cAsym space. First, a reference volume and its skull-stripped version were generated using a custom methodology of fMRIPrep. Several confounding time-series were calculated based on the preprocessed BOLD: framewise displacement (FD), and three region-wise global signals. FD was computed using two formulations following Power (absolute sum of relative motions) and Jenkinson (relative root mean square displacement between affines). FD is calculated for each functional run using its implementations in Nipype. The three global signals are extracted within the CSF, the WM, and the whole-brain masks.

Additionally, a set of physiological regressors were extracted to allow for component-based noise correction (CompCor). Principal components are estimated after high-pass filtering the preprocessed BOLD time-series (using a discrete cosine filter with 128s cut-off) for anatomical component correction (aCompCor). For aCompCor, three probabilistic masks (CSF, WM and combined CSF+WM) are generated in anatomical space. The implementation differs from that of Behzadi et al. in that instead of eroding the masks by 2 pixels on BOLD space, the aCompCor masks are subtracted from a mask of pixels that likely contain a volume fraction of GM. This mask is obtained by thresholding the corresponding partial volume map at 0.05, and it ensures components are not extracted from voxels containing a minimal fraction of GM. Finally, these masks are resampled into BOLD space and binarized by thresholding at 0.99 (as in the original implementation). Components are also calculated separately within the WM and CSF masks. For each CompCor decomposition, the k components with the largest singular values are retained, such that the retained components’ time series are sufficient to explain 50 percent of variance across the nuisance mask (CSF, WM, combined, or temporal). The remaining components are dropped from consideration. The head-motion estimates calculated in the correction step were also placed within the corresponding confounds file. All resamplings can be performed with a single interpolation step by composing all the pertinent transformations (i.e., head-motion transform matrices, susceptibility distortion correction when available, and co-registrations to anatomical and output spaces). Gridded (volumetric) resamplings were performed using àntsApplyTransforms` (ANTs), configured with Lanczos interpolation to minimize the smoothing effects of other kernels. Many internal operations of fMRIPrep use Nilearn 0.6.2, mostly within the functional processing workflow. For more details of the pipeline, see the section corresponding to workflows in fMRIPrep’s documentation (https://fmriprep.readthedocs.io/en/latest/workflows.html).

Next, we applied spatial smoothing with a 5mm full-width at half-maximum (FWHM) Gaussian kernel using FEAT (FMRI Expert Analysis Tool) Version 6.00, part of FSL (FMRIB’s Software Library, www.fmrib.ox.ac.uk/fsl). Non-brain removal using BET (Smith et al., 2002) and grand mean intensity normalization of the entire 4D dataset by a single multiplicative factor were also applied.

### Neuroimaging Analyses

Neuroimaging analyses used FSL version 6.0.4 (Jenkinson et al., 2012; S. M. Smith et al., 2004). We used two general linear models. Both types of models were based on a general linear model with local autocorrelation (Woolrich et al., 2001). Our first model focused on the brain activation evoked during the decision phase of the DG-P, UG-P, and UG-R conditions and used a total of thirteen regressors. Six regressors reflected the endowment phase (duration = 1,000 ms) across all three conditions (DG-P, UG-P, and UG-R) for both unmodulated and parametrically modulated analyses. For the parametrically modulated regressors, we calculated the proportion of the endowment proposed as the independent variable. Proportions closer to 0.5 reflected a more even split and were indicative of fairer options, whereas proportions closer to 0 reflected more unfair offers. Six regressors reflected the three task conditions during the decision phase (duration = 3,000 ms). We modelled both unmodulated and parametrically modulated effects for each task. Finally, the thirteenth regressor modelled missed trials.

Our second type of model focused on the task-dependent connectivity using the ventral striatum as a seed and areas related to social processing as target regions. To estimate the changes in connectivity resulting from strategic behavior, we used psychophysiological interaction (PPI) analysis (Friston et al., 1997; O’Reilly et al., 2012a). This form of analyzing effective connectivity has been shown through meta-analyses to reveal reliable and specific patterns of task-dependent connectivity (D. V. Smith, Gseir, et al., 2016; D. V. Smith, Rigney, et al., 2016; D. V. Smith & Delgado, 2017). Our PPI analysis focused on effective connectivity using the ventral striatum (VS; Oxford-GSK-Imanova atlas) as a seed. Additionally, we used seeds derived from whole-brain analyses to find non-pre-registered target regions in secondary analyses (O’Reilly et al., 2012b). The average time course of activation from this seed region was extracted and used as an additional fourteenth regressor. To construct the PPI model, we used the same model described above and added 14 additional regressors (1 regressor for the VS region and 13 regressors for the interaction between the VS region and the task-based regressors), yielding a total of 25 regressors in each seed-based PPI model. Both activation and connectivity models were then run through a second level analysis that combined the first and second runs. For participants with missing data, or for runs that were excluded due to head motion, we used a participant’s one good L1 run in the group level analyses. Both models also included a common set of confound regressors. We also included additional regressors for the six motion parameters (rotations and translations), the first six aCompCor components explaining the most variance, non-steady state volumes, and the framewise displacement (FD) across time. Finally, we used high-pass filtering (128s cut-off) through a set of discrete cosine basis functions.

For all participants and their combined runs, we used a fixed-effects model. These models focused on activation and connectivity patterns and their associations between bargaining behavior, substance use and BOLD responses, independent of reward sensitivity. Group-level analysis was carried out using FLAME (FMRIB’s Local Analysis of Mixed Effects) Stage 1 (Beckmann et al., 2003; Woolrich et al., 2004). Our group-level model focused on comparisons between the Dictator and Ultimatum Games as a Proposer; these comparisons included covariates to account for reward sensitivity, the second-order polynomial expansion of reward sensitivity (which captures effects tied to aberrant reward sensitivity), substance use, strategic behavior, temporal signal to noise ratio (tSNR) and mean framewise displacement (fd mean). We also used two additional models that explore interaction effects. The first interaction model included additional regressors of substance use and reward sensitivity and substance use and aberrant reward sensitivity. The second interaction model included additional regressors of the interaction of strategic behavior and reward sensitivity, and strategic behavior and aberrant reward sensitivity.

All pre-registered hypotheses were first tested with a priori regions of interest. We used the Harvard-Oxford Atlas to make masks for the dlPFC, mPFC (ie: paracingulate gyrus), SPL, ACC, and Insula. We used the Oxford-GSK-Imanova and Striatal Connectivity Atlases to define the nucleus accumbens mask. Next, we used the Mars TPJ Atlas for our right pTPJ mask. Finally, for the vmPFC mask, we used the Clithero and Rangel, 2013 meta-analysis coordinates (−2, 28, −18) and a 5mm sphere. Each pre-registered ROI-based analysis was followed by a whole-brain analysis to minimize Type II errors. All z-statistic images were thresholded and corrected for multiple comparisons using an initial cluster-forming threshold of z > 3.1 followed by a whole-brain corrected cluster-extent threshold of p < 0.05, as determined by Gaussian Random Field Theory (Cao & Worsley, 2001; Eklund et al., 2016; Flandin & Friston, 2019; Nichols & Hayasaka, 2003). Secondary, and exploratory whole brain analyses reported coordinates using the center of gravity (CoG).

### Deviations from Pre-Registration

Once we began data collection and analyses, we made several adjustments based on four issues that were unspecified in our pre-registration. First, we initially specified that we would use the parametric effect of endowment, but not for decisions. For decisions, we expected to use the actual offers selected (High, Low) in our analyses. However, since many participants selected High more often in the UG condition and Low in the DG condition, these regressors had fewer events for comparison. To address this issue, we modeled strategic decisions as parametric effects of offer amount through the difference in the proportions of the endowments offered between DG-P and UG-P. Second, we adjusted the covariates in our group level models due to missing data. Although we originally planned to study Machiavellianism, due to an error in data collection, this survey was not completed by our participants. Next, whereas substance use analyses were not mentioned in the pre-registration, we intended to complete them in accordance with the broader aims and hypotheses of the grant, which are also described in the grant report (Sazhin et al., 2020). Third, we used the (Clithero & Rangel, 2014) (−2, 28, −18) meta-analysis vmPFC coordinates for our mask rather than the mask specified in the pre-registration (Delgado et al., 2016) for greater spatial specificity in our analyses. Fourth, we explored group level models that included the interaction of reward sensitivity, substance use and strategic behavior despite not being initially pre-registered. Taken together, these adjustments from the pre-registration have allowed us to analyze the data more robustly, though our results and discussion take greater care to differentiate between confirmatory and exploratory results, especially emphasizing differences in our group level models.

## Results

Below, we report results from behavioral analyses, task-based neural activation and connectivity analyses, followed by secondary and exploratory whole-brain analyses. We begin by presenting results of the behavioral tasks, assessing whether participants made choices as expected, and their self-reported levels of emotional intelligence, attitudes toward rejection, reward sensitivity, and substance use. Next, we present both pre-registered region of interest and secondary whole-brain analyses activation results during the endowment and decision phases. We then present results of pre-registered, secondary and exploratory psychophysiological interaction (PPI) analyses examining strategic choices between the dictator and ultimatum games within reward-related and social neural systems. Finally, we present associations between attitudes toward fairness, reward sensitivity, and brain connectivity.

### Strategic Behavior

**Figure 2.**
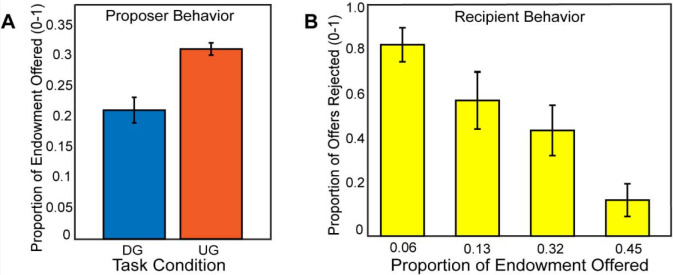
Participants make strategic decisions by offering lower in the Dictator Game versus the Ultimatum Game. In Panel A, we find that participants made higher offers in the Ultimatum Game as a proposer compared to the Dictator Game. In Panel B, we show that participants rejected unfair offers more frequently when they acted as a recipient in the Ultimatum Game. Overall, these behavior results are consistent with our hypotheses and past literature.

If participants made higher offers in the Ultimatum Game compared to the Dictator Game, this would indicate that participants were acting most consistently toward maximizing their earnings, thereby exhibiting strategic behavior. Consistent with our expectations, using a mixed effects model for a random intercept, we found that participants (N=54) made more selfish offers in the DG vs. the UG conditions, (B = −0.43, SE = 0.015, *t*(2550) = −28.09, *p* <.001), (see Figure 2). As a manipulation check, we investigated whether participants rejected unfair offers in the recipient condition. A binary logistic regression indicated that participants reject more often with lower offers, (B = 1.72, SE = 0.095, *t*(1252)= 18.06, *p* <.001). Next, we explored whether there was a relation between strategic behavior and rejection rate as a function of offer amount as a recipient, finding no significant association, *r*(52) = −.19, *p* =.16. Given that there was no relationship of recipient choices to strategic decisions as proposers, we excluded these measures from subsequent analyses.

Next, we assessed whether measures of social functioning (N=45) were related to strategic decisions. Several participants had missing questionnaire data, resulting in a smaller dataset for these analyses. Consistent with our hypotheses, individuals scoring higher on the Emotional Intelligence (EI) scale made higher offers as a proposer in the Ultimatum Game, *r*(43) = .35, *p* = .02. Contrary to our hypotheses, we did not find associations between strategic behavior, emotional intelligence, or attitudes toward rejection that met a *p-*value of less than *p*=.05. Inasmuch as there was no effect of strategic behavior and our measures of social functioning as we hypothesized, we excluded these measures from further analyses and used the full dataset of 54 participants for further analyses.

Although we did not expect relations between strategic behavior and measures of reward sensitivity and substance use, we explored whether there were such associations to contextualize any brain relations we may have found with these respective individual difference measures. We did not find any significant associations between reward sensitivity and substance use, and strategic behavior or individual task conditions (DG-P, UG-P, UG-R) that met a threshold of *p* < .05.

### Neural Responses during Endowment

Our first goal for our neuroimaging analyses was to examine the response to endowment. We hypothesized that responses in the ventral striatum would increase as a function of the endowment size. We conducted an ROI-based confirmatory analysis, expecting activation in the VS and the vmPFC. We expected that VS and vmPFC responses would vary based on the expectation of how much of the endowment would subsequently be kept in the decision phase, with the greatest amounts in DG-P, moderate in UG-P, and lowest in UG-R. Contrary with our hypotheses, we did not find a significant difference in vmPFC activation during the endowment phase between the DG-P, UG-P, and UG-R conditions, using a one-way ANOVA (2,159) *F* = 2.40 *p* = 0.09. Next, we did not find significant activation in the VS during the endowment phase when we used a one-way ANOVA to compare activation between the DG-P, UG-P, and UG-R task conditions *F*(2,159) = 1.10 *p* = 0.34 as participants received greater proportions of the endowment.

Next, we investigated whether there were associations between reward sensitivity, neural activation, and the levels of endowment presented. We expected that the response in the VS and vmPFC would positively vary based on the endowment. Such an association would reveal if there were moderating variables in VS or vmPFC activation as participants are endowed with higher levels of money when there is a threat of rejection (UG) versus when there is not (DG). Neither confirmatory ROI-based analyses nor secondary whole-brain analyses found an association between the level of activation in the vmPFC or VS, amount of endowment, and substance use or reward sensitivity.

### Neural Responses while Making Strategic Decisions

To examine the effect of bargaining decisions, we initially conducted confirmatory analyses to investigate how the brain responds as people exhibit strategic behavior through offering fairer offers in the Ultimatum Game (UG) versus the Dictator Game (DG). We hypothesized that we would find activation in areas previously examined (Spitzer et al., 2007), and brain areas associated with social processing, specifically the Anterior Cingulate Cortex (ACC), Superior Parietal Lobule (SPL), and posterior temporoparietal junction (rpTPJ). We conducted a ROI analysis in each of these regions and we did not find any significant activation that met *p* = .05 or lower.

We followed up with secondary whole brain analyses with appropriate adjustments for multiple comparisons to investigate whether there are other regions that may reflect strategic decision making. When assessing how people chose to be selfish versus fair in the contrast between the DG and UG as a proposer, we found significant clusters in the Inferior Frontal Gyrus (IFG) (MNIxyz = 51, 24, 24; cluster = 20 voxels, *p*=.035) and a cluster spanning the Anterior Insula (AI), extending into the Orbitofrontal Cortex (OFC) (MNIxyz = 33, 27, −4; cluster = 54 voxels, *p*<.001). We did not find significant activation in the vlPFC or the VS. In the contrast between UG and DG (i.e., choosing to be fair versus unfair), we found a significant cluster in cerebellum (MNIxyz = 30,−82, −36; cluster = 37, *p*<.001). In sum, some of our results successfully replicated past investigations of strategic behavior.

### Strategic Behavior and Neural Connectivity

Beyond activation patterns, we studied whether task-dependent connectivity patterns related to reward sensitivity and strategic decisions made in the Dictator and Ultimatum games. First, we conducted confirmatory analyses to examine our pre-registered ROI-based hypotheses using the ventral striatum as a seed. During the decision phase for the proposer conditions, we expected to find that ventral striatal responses to situations requiring strategic thinking (UG-P > DG-P) would be associated with enhanced connectivity with regions modulated by social information. To test this hypothesis, we conducted a PPI analysis using the VS as a seed region, and we focused on connectivity with a priori target regions (vmPFC, mPFC as defined by paracingulate gyrus, rpTPJ, and the SPL). Contrary to our hypotheses, we did not find any significant connectivity between a VS seed and our expected regions of interest that survived multiple comparisons.

Next, we conducted secondary whole-brain analysis to assess relations of other possible target regions to reward sensitivity and strategic behavior. We included the IFG and AI as seeds because they were derived from the activation of DG versus UG contrast in our data. Our group level analyses employed several covariates, including motion-based nuisance regressors, reward sensitivity, substance use, and strategic behavior. We also used two additional models that investigated the interactions of reward sensitivity, strategic behavior, and substance use respectively.

**Figure 4.**
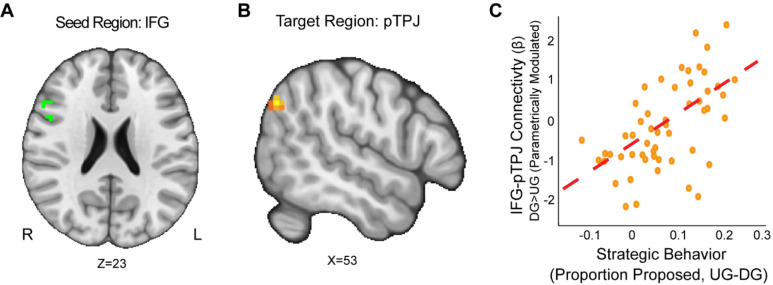
IFG-rpTPJ Connectivity is Modulated by Strategic Behavior. We found that connectivity between an Inferior Frontal Gyrus (IFG) seed (Panel A), and a right pTPJ target (Panel B) was related to elevated strategic behavior (Panel C) (DG > UG) (MNIxyz = 50, −68, 35; cluster = 22 voxels, p = .008).(Thresholded: https://neurovault.org/images/803475/ Unthresholded https://neurovault.org/images/803476/). These results suggest that IFG- right pTPJ connectivity may modulate strategic behavior contingent on whether there is a threat of rejection or not. Participants who experienced elevated IFG-right pTPJ connectivity were also those who were more selfish in DG and offered closer to even splits in UG. For illustrative purposes, we extracted the parameter estimates within each region (Panel C). We note that Z statistic images were thresholded parametrically (Gaussian Random Field Theory) using clusters determined by Z > 3.1 and a (corrected) cluster significance threshold of p=.05 and the images are plotted using radiological view.

First, we wanted to examine if strategic behavior as measured by the choices our participants made was associated with brain connectivity. Using the IFG as a seed (MNIxyz = 52, 16, 22), we found that enhanced connectivity with a left rpTPJ target region (Schurz et al., 2017) extending into the SMG (MNIxyz = 50, −68, 35; cluster = 22 voxels, *p* = .008) was modulated by strategic behavior in the Dictator versus Ultimatum Game (see Figure 4). That is to say, selfish participants (i.e.: by making lower proposals in the DG versus UG conditions) experienced enhanced IFG-rpTPJ connectivity contingent on whether or not there was a threat of rejection. Our results suggest that enhanced IFG-rpTPJ connectivity may facilitate the social processing associated with strategic decisions in social contexts. We also examined if connectivity from an AI seed related to strategic situations was modulated by strategic behavior. Using the AI seed (MNIxyz = 33, 27, −4), we found that attenuated connectivity with the neighboring insular cortex (MNIxyz = 50, 6, −1; cluster = 26 voxels, *p* = .003) was modulated by strategic behavior in UG versus DG condition. That is to say, participants who were more selfish when there was no threat of rejection exhibited lower AI-Insula connectivity. Our results suggest that attenuated co-activation of the insular cortex may contribute to making more selfish choices in social contexts.

### The Association Between Connectivity with the Anterior Insula-Angular Gyrus and Trait Reward Sensitivity is Modulated by Strategic Behavior

Next, we examined how the interaction of reward sensitivity and substance use may modulate brain connectivity patterns associated with strategic thinking in bargaining situations. Investigating how a trait like reward sensitivity may modulate brain responses can reveal an important factor for understanding both behavior and brain relationships. Specifically, we used a model that included interaction covariates of strategic thinking with reward sensitivity and aberrant reward sensitivity. The model also controlled the main effects of strategic behavior, reward sensitivity, aberrant reward sensitivity, and substance use. We included substance use as a controlling variable due to its known relationships with reward sensitivity in psychopathology (Joyner et al., 2019).

**Figure 5.**
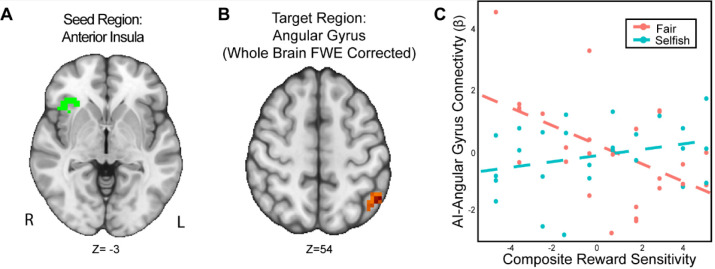
The interaction of reward sensitivity and strategic behavior modulated AI – Angular Gyrus connectivity in social situations requiring strategic thinking. We conducted a whole-brain analysis exploring the interaction of trait reward sensitivity and strategic behavior. We found that higher reward sensitivity is associated with 1) more strategic behavior and 2) elevated effective connectivity between AI (Panel A) and the Angular Gyrus (MNI; xyz = −47,−56, 54; cluster = 23 voxels, p = .005). Conversely, for participants with low reward sensitivity, we found that their AI-Angular connectivity is lower as they exhibit strategic behavior. For illustrative purposes (Panel C), we used a median split to indicate the relation of reward sensitivity and strategic behavior. Next, we extracted the parameter estimates within each region (Panel C). We note that Z statistic images were thresholded parametrically (Gaussian Random Field Theory) using clusters determined by Z > 3.1 and a (corrected) cluster significance threshold of p=.05 and the images are plotted using radiological view. See images here:(Thresholded: https://neurovault.org/images/803477/; Unthresholded: https://neurovault.org/images/803482/).

We found that the interaction of reward sensitivity and strategic behavior modulated AI-Angular Gyrus connectivity in the UG versus DG condition (Figure 5). That is to say, participants with higher reward sensitivity and attenuated AI-Angular Gyrus connectivity tended to make more strategic choices when there was a threat of rejection relative to when there was not. Moreover, participants with lower reward sensitivity *and* enhanced AI-Angular Gyrus connectivity tended to make more strategic choices when there was a threat of rejection compared to when there was not. These results suggest that the combination of strategic decisions and a person’s trait reward sensitivity together may modulate connectivity patterns in social situations requiring strategic thinking.

## Discussion

This study investigated how relations between strategic behavior in bargaining situations and reward responses correspond to patterns of brain activation and connectivity. First, our behavioral results are consistent with past work suggesting that participants act strategically in bargaining situations through acting fairly when there is a threat of rejection (e.g., Ultimatum Game; UG) while keeping more for themselves when there is not a threat of rejection (Dictator Game; DG) (Charness & Gneezy, 2008). Second, we found that strategic behavior between the Dictator and Ultimatum Games evoked activation in the inferior frontal gyrus (IFG) and Anterior Insula (AI), results that were consistent with past investigations (i.e., Spitzer et al., 2007). Our analyses also indicated that elevated IFG-rTPJ connectivity was related to enhanced strategic behavior and attenuated AI-Angular Gyrus connectivity was modulated by the interaction of reward sensitivity and strategic behavior.

Our work fits in with past literature suggesting that norm compliance is regulated by cortical activation. Although we did not find activation during UG versus DG in our pre-registered regions of interest, our whole brain analyses revealed activation in the right IFG and AI as participants made strategic decisions, replicating previous work (Spitzer et al., 2007; Zheng & Zhu, 2013). Next, both IFG and AI activation has been observed in other decision-making contexts. For example, FeldmanHall and colleagues reported AI activation during moral decision making (FeldmanHall et al., 2014). In addition, other work has shown that increased activation in the anterior insula in a trust task is associated with inequity aversion (van Baar et al., 2019; FeldmanHall et al., 2014). Further, our results are consistent with stimulation-based research that found elevated right dlPFC area activation corresponded to more strategic behavior (Knoch et al., 2006; Ruff et al., 2013; Strang et al., 2015) and inhibition of dlPFC activity diminished strategic choices (Müller-Leinß et al., 2018; Zinchenko et al., 2021). In sum, our findings are consistent with the IFG and AI being involved in norm compliance decisions.

Our work extends past literature through investigating how reward processes and cortical connectivity interact with strategic behavior. Our results indicate that elevated IFG-rpTPJ connectivity is associated with increased strategic behavior, whereas attenuated AI-Angular Gyrus connectivity is modulated by the interaction of reward sensitivity and strategic behavior. Although recent work has shown that the dlPFC and rpTPJ regulate norm compliance in the UG and DG (Gianotti et al., 2018), and that the right TPJ does not necessarily yield greater generosity (Brethel-Haurwitz et al., 2022), our results indicate that the connectivity between these brain regions modulates strategic decisions in social situations. Understanding how connectivity modulates strategic decisions is a critical component of characterizing how the TPJ and dlPFC may be regulated during decision making. Nonetheless, when including reward sensitivity as a covariate, we find that this relationship changes as people with low reward sensitivity are more strategic with decreasing AI-Angular Gyrus connectivity.

It has been previously found that reward sensitivity is associated with risky behavior (Scott-Parker & Weston, 2017), higher Machiavellianism (Birkás et al., 2015), and with greater strategic behavior (Scheres & Sanfey, 2006). Since the angular gyrus is implicated in bottom-up attentional processing (Seghier, 2013), we speculate that reward sensitivity may modulate strategic decisions through increasing attention to changes in social context toward maximizing earnings. Additionally, among the many functions of the AI includes integrating fairness, empathy, and cooperation (Cheng et al., 2017; Lamm & Singer, 2010), which may suggest that AI-Angular Gyrus connectivity could orient participants toward changes in context affecting norms. Thus, AI-Angular Gyrus connectivity may be modulated by reward sensitivity through processing opportunities to cooperate and defect, which could affect how people experience social heuristics or empathy toward others in bargaining situations.

We speculate that people with higher reward sensitivity may be more motivated to closely examine their choices, and thus, may be more likely to defect in bargaining tasks. Increased deliberation may in turn override default empathetic processes in determining what is fair to others. Overall, our results suggest that to understand strategic decisions, we may need to examine psychosocial factors such as reward sensitivity to investigate how they modulate strategic decision making. As such, our results suggest a nuanced view of AI-Angular Gyrus and IFG-TPJ coupling (Lockwood et al., 2020), indicating that these brain regions do not necessarily reflect altruistic choice on their own (Hutcherson et al., 2015), but may modulate cognitive reward processes while making social decisions. We speculate that our results suggest the downregulation of bilateral TPJ activation and AI deactivation (FeldmanHall et al., 2014) interacts with trait reward sensitivity.

Although our work has found that strategic behavior is modulated by both AI-Angular Gyrus and IFG connectivity with the TPJ, and reward sensitivity, we acknowledge that our study has several limitations that merit discussion. First, although we found relations with bilateral TPJ connectivity and strategic behavior, we do not infer specificity in lateralization. Past investigations suggest mixed findings (Carter et al., 2012; Coricelli & Nagel, 2009; Saxe & Kanwisher, 2003) as to the roles of the right and left TPJ, and we judged that exploring these results further was beyond the scope of our paper. Second, we acknowledge that conducting brain-wide association tests with individual difference measures may be underpowered (Marek et al., 2022) and that multivariate methods can improve effect size estimation in neuroimaging (Reddan et al., 2017). Although we initially sampled from people with high and low reward sensitivity and conducted rigorous test-retest with SR and BIS/BAS to ensure that participants were consistent across these measures, we acknowledge that relations with reward sensitivity should be considered exploratory in nature. Future analyses could use multivariate methods such as canonical correlation analysis (Zhuang et al., 2020), multivariate pattern analysis (Kragel & LaBar, 2015) or use machine learning algorithms to assess neural signatures (Wager et al., 2013) of bargaining to further examine how reward sensitivity modulates brain responses.

Third, we note that relations with social context, reward sensitivity, and brain connectivity could be studied more extensively in a clinical population to assess how these relations are modulated by substance use and manic-depressive symptoms. Whereas we were able to control for levels of substance use to account for reward sensitivity effects (Joyner et al., 2019), we had too limited variability in substance use to make inferences about how substance use may contribute to maladaptive strategic decisions. Fourth, we acknowledge that connectivity analyses are not causal or directional with respect to inference despite identifying the IFG and AI as seeds and the temporoparietal junction as target. Another possible future direction includes evaluating AI-Angular Gyrus and IFG-TPJ connectivity patterns, associations with reward sensitivity, and their relations with recipient decisions in the Ultimatum Game. Fifth, since strategic behavior as a proposer was not related to recipient choices, we judged that these results are beyond the scope of this investigation. Finally, while we assessed strategic behavior, we did not assess it in a dynamic context. As social contexts increase exploration and obtained rewards (Plate et al., 2023), a fruitful future direction could investigate how brain responses to changes over time reflect social decisions.

Despite the limitations, our findings indicate that strategic decisions in social contexts are associated with AI-Angular Gyrus and IFG-TPJ connectivity and are modulated by trait reward sensitivity. These results provide greater insights into how reward processes interact with social decisions, involving brain processes that appraise the roles of other people while making choices. Since aberrant reward sensitivity is a major mechanism in substance use and depressive and bipolar disorders, investigating how reward sensitivity modulates brain processes during social contexts could provide considerably more understanding into how people make maladaptive decisions resulting in substance use (Bart et al., 2021; Heilig et al., 2016; Wyngaarden et al., 2023). Such work could help identify people at risk for substance use disorders and help develop interventions for people with aberrant reward patterns.

## Figures and Tables

**Figure 3.**
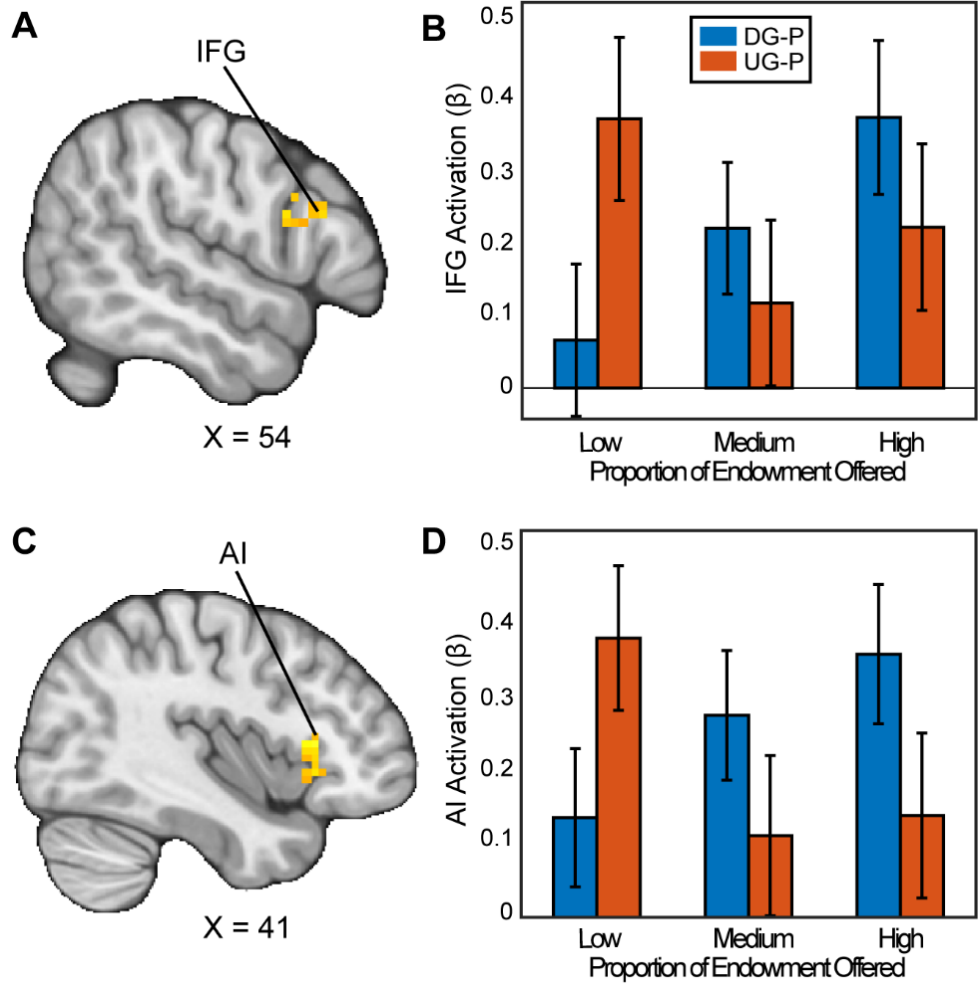
Activation associated with strategic thinking. We conducted a whole-brain analysis to investigate whether there were regions in the brain that differentially responded when acting as a proposer in the DG versus UG conditions. When assessing the parametric effect associated with acting more strategically, Panels A and C reflect regions (Inferior Frontal Gyrus (IFG) (MNIxyz = 52, 16, 22; cluster = 20 voxels, p=0.035, and Anterior Insula (AI) extending into the Orbitofrontal Cortex (OFC) (MNIxyz = 37, 23, 2; cluster = 54 voxels, p<.001 respectively) with significant activation. That is to say, as participants made fairer offers in the DG condition, they experienced stronger activation compared to when they made fairer offers in the UG condition. (Thresholded: https://neurovault.org/images/803473/; Unthresholded: https://neurovault.org/images/803474/). For illustrative purposes, Panels B and D shows the extracted parameter estimates within each region. We note that Z statistic images were thresholded parametrically (Gaussian Random Field Theory) using clusters determined by Z>3.1 and a (corrected) cluster significance threshold of p=.05.

**Table 1:** We incorporated several group level models assessing strategic behavior and reward sensitivity while controlling for substance use. We assessed the interactions of reward sensitivity and strategic behavior and substance use respectively. If there were no interaction effects, we interpreted main effects using the no interaction model. We completed these group level analyses across both activation and PPI models. The PPI model used a pre-registered VS seed, and IFG and AI seeds as derived from our secondary whole-brain results. The initial group level models were derived from initial hypotheses, though the interaction of reward sensitivity and strategic behavior was an exploratory model driven by our results. Thresholded and unthresholded images are available on Neurovault: https://neurovault.org/collections/15045/

Model Type	Confirmatory/Exploratory	Covariates
No Interactions	Confirmatory	Strategic Behavior, Substance Use, Reward Sensitivity, Aberrant Reward Sensitivity
Reward Sensitivity × Substance use	Confirmatory	No Interaction model plus substance use × reward sensitivity, substance use × aberrant reward sensitivity
Reward Sensitivity × Strategic Behavior	Exploratory	No Interaction model plus strategic behavior × reward sensitivity, strategic behavior × aberrant reward sensitivity

## Data Availability

Analysis code related to this project can be found on GitHub: (https://github.com/DVS-Lab/istart-ugdg). Thresholded and unthresholded statistical maps are located on https://neurovault.org/collections/15045/. In addition, all data will be made available on OpenNeuro before publication.
